# Successful anatomical closure of a photographically documented 30-year-old idiopathic full-thickness macular hole following surgery for concurrent repair of an acute macula-on rhematogenous retinal detachment

**DOI:** 10.1093/jscr/rjae231

**Published:** 2024-04-14

**Authors:** Jonathan T W Au Eong, Jason H M Lim, Sachin M George, Kah-Guan Au Eong

**Affiliations:** Lee Kong Chian School of Medicine, Nanyang Technological University, 11 Mandalay Road, Singapore 308232, Singapore; Booragoon Eye Clinic, Unit 13, 173 Davy Street, Booragoon, WA 6154, Australia; Chaithanya Eye Institute, NH Ln, Sonia Nagar, Palarivattom, Kochi, Kerala 682024, India; Lee Kong Chian School of Medicine, Nanyang Technological University, 11 Mandalay Road, Singapore 308232, Singapore; International Eye Cataract Retina Centre, Mount Elizabeth Medical Centre and Farrer Park Medical Centre, 1 Farrer Park Station Road #14-07/08, Farrer Park Medical Centre, Connexion, Singapore 217562, Singapore; Department of Ophthalmology and Visual Sciences, Khoo Teck Puat Hospital, 90 Yishun Central, Singapore 768828, Singapore

**Keywords:** macular hole, macular hole surgery, retinal detachment, vitrectomy, vitreoretinal surgery

## Abstract

A 62-year-old man with a 30-year-old photographically documented idiopathic full-thickness macular hole and visual acuity of 6/45 developed an acute macula-on rhegmatogenous retinal detachment in his left eye. A pars plana vitrectomy, internal limiting membrane peeling around the macular hole, fluid-air exchange, endolaser retinopexy around the peripheral retinal break and perfluoropropane (C3F8) internal tamponade were performed to repair the detached retina and macular hole. One month postoperatively, the patient developed a large peripheral circumferential retinal tear with shallow retinal detachment which necessitated scleral buckling, repeat vitrectomy, endolaser photocoagulation and C3F8 tamponade. The retina was successfully re-attached and the macula hole was closed. Three years post-vitrectomy, the repaired 30-year-old macular hole remained closed although the visual acuity remained unchanged at 6/45. In summary, we describe the successful anatomical closure of a 30-year-old idiopathic full-thickness macular hole which we believe to be the longest duration photographically documented macular hole closed following surgery.

## Introduction

Macular holes are retinal defects affecting the central part of the fovea and can be idiopathic or traumatic in origin [1]. Idiopathic full-thickness macular holes are visually disabling and occur bilaterally in 10% of cases [2]. Many surgeons believe that long-standing macular holes are less likely to close successfully with surgery [3] and some are reluctant to operate on them. To the best of our knowledge, the longest standing macular hole which underwent vitreoretinal surgery was a 29-year-old hole in Kelly and Wendel’s series [4]. There was no anatomical closure of the macular hole postoperatively. In this report, we present what we believe to be the longest duration photographically documented macular hole closed successfully following surgery.

## Case report

**Figure 1:**
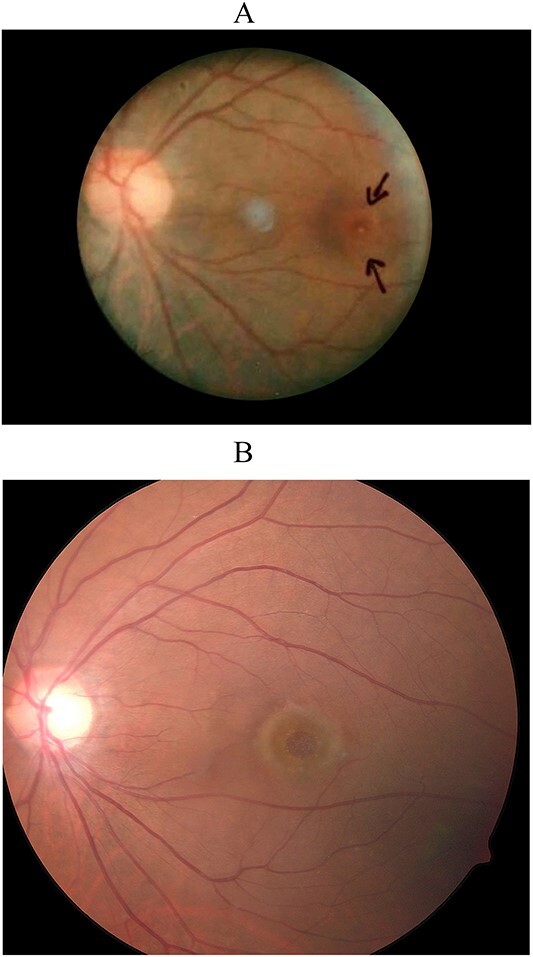
Color fundus photographs of the patient’s left eye taken 30 years earlier (A) and before surgery (B) showing the patient’s full-thickness macular hole.

A 62-year-old man with bilateral idiopathic full-thickness macular holes was referred by his ophthalmologist for macular hole surgery. His visual acuity was 6/21 in the right eye and 6/45 in the left eye. He was first diagnosed with a full-thickness macular hole in his left eye which was photographically recorded 28 years earlier (Fig. 1). The macular hole in the right eye was less than one year in duration.

We repaired the right macular hole successfully by pars plana vitrectomy, internal limiting membrane peeling and perfluoropropane (C_3_F_8_) intraocular gas tamponade. At follow-up visits over the year, the hole remained closed and his visual acuity improved to 6/6. As the visual prognosis for surgical repair of the macular hole in his left eye was guarded, the patient declined surgery for his left eye.

Two years later, the patient complained of a four-day history of an inferior visual field defect in his left eye. His visual acuity was unchanged at 6/45. Clinical examination disclosed a macula-on superonasal rhegmatogenous retinal detachment with a single large U-shaped retinal tear. Left retinal detachment surgical repair was thus arranged. In consenting the patient for the procedure, the option to repair the co-existing macular hole was discussed since we were already planning on performing pars plana vitrectomy, endolaser photocoagulation and C_3_F_8_ internal tamponade to repair the detached retina. The patient agreed and we proceeded to repair the detached retina and the full-thickness macular hole of 30 years duration.

Intraoperatively, a complete pars plana vitrectomy, internal limiting membrane peeling around the macular hole, fluid-air exchange and laser retinopexy around the peripheral retinal break were performed. C_3_F_8_ 14% was injected before the sclerotomies and conjunctiva were closed.

Postoperatively, the patient was advised to posture prone with alternate cheek to pillow every two hours for the first day. Subsequently, he postured straight face-down alternating with left cheek to pillow every few hours for 2 weeks. On follow-up visits, the peripheral retina was re-attached and the peripheral retinal tear remained closed as did the macular hole. However, one month postoperatively, the patient developed a large temporal peripheral circumferential retinal tear with shallow retinal detachment which necessitated scleral buckling, repeat pars plana vitrectomy with endolaser photocoagulation and C_3_F_8_ tamponade. Thereafter, the retina was successfully reattached.

**Figure 2:**
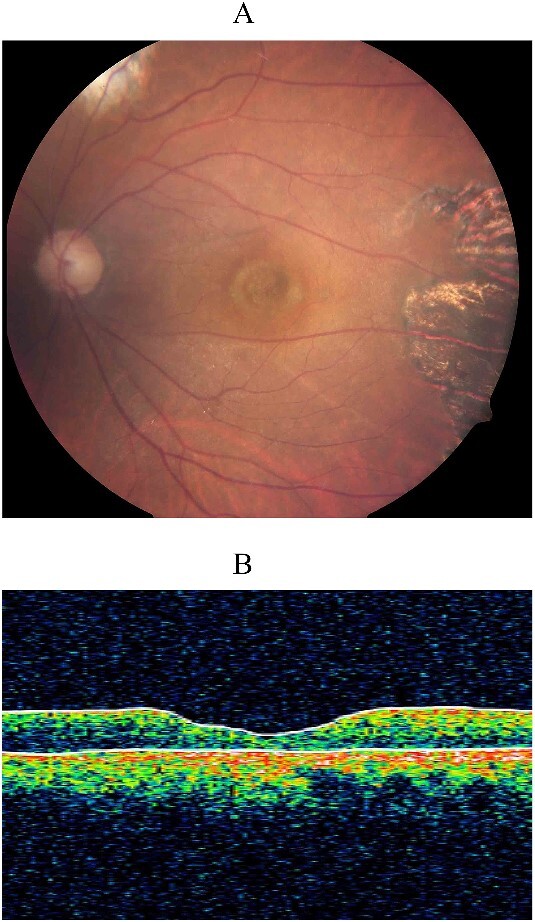
Color fundus photograph (A) and optical coherence tomography (OCT) (B) 3 years post-vitrectomy showing sustained closure of the surgically repaired 30-year-old macular hole. The chorioretinal scarring in the temporal macula was due to laser retinopexy during his second vitreoretinal surgery.

Three years post-vitrectomy, the repaired 30-year-old full-thickness macular hole remained closed (Fig. 2), with the retina remaining attached without further retinal breaks. His visual acuity in the left eye, however, remained stable at 6/45.

## Discussion

While it is widely known that the sooner macular holes are repaired the better the visual and anatomical outcome, it has also been documented that surgery for chronic macular holes can also have anatomical and visual success, although the success rates are not as high as in acute macular hole surgical repair [5]. Nonetheless, successful anatomical closure of macular holes may help to prevent further visual acuity deterioration [6].

In an individual participant data meta-analysis of 12 randomized controlled trials involving 940 eyes with a primary hole closure rate of 81.5% by Murphy *et al*. [2] multilevel logistic regression showed that each additional month of macular hole duration was associated with 0.965 times lower odds of hole closure (95% confidence interval [CI], 0.935–0.996, *P* = 0.026). For eyes which achieved primary hole closure, multilevel logistic regression showed that each additional month of symptom duration was associated with worsening best-corrected visual acuity by 0.008 logarithm of the minimum angle of resolution (logMAR) units (95% CI, 0.005–0.011, *P* < 0.001) or about one Early Treatment Diabetic Retinopathy Study letter loss per 2 months [2]. On the basis of these results, the authors advocated prompt referral and surgery for all macular holes as the best means of achieving macular hole closure and good final functional results [2].

In summary, despite a lack of improvement in visual acuity in our patient post closure of the macular hole, he did achieve anatomical closure of what we believe to be the longest photographically documented macular hole of 30 years duration. More studies to investigate the anatomical and functional outcomes of macular hole surgery on very long-standing macular holes are warranted so that surgeons can weigh the potential benefits and risks of surgery in such cases.
